# Inflammatory biomarkers as predictors of prognosis in patients after transcatheter aortic valve implantation

**DOI:** 10.3389/fcvm.2025.1722293

**Published:** 2026-01-02

**Authors:** Luka Vitez, Peter Marko Mihailović, Mojca Božič Mijovski, Borut Jug, Matjaž Bunc

**Affiliations:** 1 Department of Cardiology, University Medical Centre Ljubljana, Ljubljana, Slovenia; 2 Faculty of Medicine, University of Ljubljana, Ljubljana, Slovenia; 3 Department of Vascular Diseases, University Medical Centre Ljubljana, Ljubljana, Slovenia

**Keywords:** inflammatory biomarkers, systemic inflammatory response syndrome, transcatheter aortic valve implantation, high sensitivity CRP (hs-CRP), interferon gamma (IFN-γ)

## Abstract

**Background:**

The expanding role of transcatheter aortic valve implantation (TAVI) highlights the need to identify factors influencing long-term outcomes. Systemic inflammatory response syndrome (SIRS) is a frequent post-procedural event that may adversely affect prognosis. Measurement of inflammatory biomarkers may improve the understanding of underlying mechanisms and refine patient risk stratification.

**Methods:**

This single-center, prospective cohort study, enrolled 62 consecutive patients undergoing TAVI, who were followed for up to 5 years. Blood samples were collected before TAVI, at 24 h and 3–6 months post-procedure. Changes in biomarker levels, predictors of SIRS, and inflammatory predictors of long-term outcomes were analyzed.

**Results:**

SIRS developed in 45% of patients. Significant temporal changes were observed in hs-CRP, TNF-α, sST2/IL-33, IL-10, and IL-2 levels, irrespective of baseline or procedural characteristics. The development of SIRS was associated with a higher risk of all-cause mortality or unplanned hospitalization at 5 years (HR 3.07, 95% CI 1.57–6.00; *p* = 0.001). Baseline hs-CRP (HR 1.21, 95% CI 1.09-1.35; *p* < 0.001) and IFN-γ (HR 1.22; 95% CI 1.09–1.36; *p* < 0.001) levels were predictive of adverse outcomes. In multivariable Cox analysis, these associations remained, though findings should be interpreted cautiously given the limited sample size.

**Conclusions:**

SIRS is a common post-TAVI phenomenon and may be linked to long-term outcomes. Elevated pre-procedural hs-CRP and IFN-*γ* levels were associated with higher risk for adverse events, suggesting they may serve as exploratory biomarkers for risk stratification in this population.

## 1 Introduction

Transcatheter aortic valve implantation (TAVI) is now a well-established treatment for severe aortic valve dysfunction in patients over 70 years of age (1). Following favorable clinical outcomes, its indications have progressively expanded to include intermediate- or even low-risk patients (2–5). With the growing number of procedures worldwide, increasing attention is being directed towards understanding the mechanisms and consequences of TAVI on patient outcomes and prognosis.

Systemic inflammatory response syndrome (SIRS) is a frequent post-procedural phenomenon, affecting approximately one third of patients (35-56%) after TAVI (6–8). This immune response is thought to result from hemodynamics changes and tissue ischemia with subsequent reperfusion injury during the procedure, potentially influencing both short- and long-term outcomes (6, 7, 9). Elevated numbers of inflammatory biomarkers—such as high sensitivity C-reactive protein (hs-CRP), leukocyte count, proinflammatory cytokines (e.g., IL-6), T-helper (Th) cells, and biomarkers of myocardial damage— have been associated with increased all-cause mortality after TAVI (7, 9, 10). While a better understanding of inflammatory pathways in SIRS following TAVI may improve prognostic assessment and patient selection, novel biomarkers could serve as promising tool for risk stratification and, potentially, future targeted therapies.

Tumor necrosis factor alpha (TNF-α) and interleukin 1 beta (IL-1β) are non-specific markers of systemic inflammation. TNF-α blockade has shown harmful effects in heart failure, suggesting elevated TNF-α may be compensatory, whereas IL-1β inhibition reduced cardiovascular events post-myocardial infarction, indicating a deleterious role in atherosclerosis (11, 12). Interleukin 2 (IL-2) and 10 (IL-10) represent more specific pro- and anti-inflammatory responses, influencing macrophage and T cell activation with *in vitro* and *in vivo* studies suggesting modulation by angiotensin-converting enzyme inhibitors (13). Interferon gamma (IFN-γ) is central to adaptive and innate immunity, with elevated levels linked to various cardiac diseases and atherosclerosis (14, 15). Soluble suppression of tumorogenesis 2 (sST2), the IL-33 receptor, is involved in cardiac remodeling and may reflect reduced antifibrotic IL-33 signaling, serving as a potential heart failure marker (16). The effects of these inflammatory indicators after TAVI have not yet been tested and their clinical value therefore remains elusive.

Our aim was to investigate these novel biomarkers by analyzing their temporal dynamics, predictive value, and association with long-term prognosis in patients undergoing TAVI.

## 2 Methods

### 2.1 Study population

This was a prospective single center study of consecutive patients referred for TAVI from July 2019 to November 2023 at the University Medical Centre Ljubljana, Slovenia. We prospectively included 70 patients that were eligible for TAVI by the local Heart team. Patients with unstable or recent cardiovascular event (<3 month prior to inclusion), acute illness or recent (<3 months prior to inclusion) non-cardiovascular disease requiring hospitalization, stage 5 chronic kidney disease, active malignancy or autoimmune disease were excluded from the study.

TAVI procedures were done via transfemoral, transapical or transaortic approach using contemporary balloon expandable (Sapien 3, Edwards, USA; Myval, Merill, India) and self-expandable (Evolute and Evolute Pro, Medtronic, USA; Portico, Abbott, USA) transcatheter aortic valves. Valve-in-valve patients were not excluded from the analysis. All procedures except transapical and transaortic were done in conscious sedation and prophylactic antibiotic therapy with three doses of second generation cephalosporins were given.

We defined SIRS according to the current guidelines (17). Patients categorized with SIRS needed to have at least two of the following criteria in the first 48 h after TAVI: leucocyte count >12.0 or <4.0 × 10^9^ /L, heart rate >90 beats per minute, respiratory rate >20 breaths per minute or PaCO2 ≤ 4.3 kPa/32 mmHg and temperature >38.0°C or <36.0°C. Procedural and clinical endpoints were defined according to the pre-defined VARC-3 criteria (18). Clinical impact of SIRS was assessed as need for any unplanned hospitalization or death for any cause (whichever came first) until the end of the prospective observation period (minimum 1 year for up to 5 years). Follow-up was carried out after 3 to 6 months, followed by a routine 1–2 years ambulatory visits or telephonic contact.

The study was approved by the National Ethics Committee (reference number: 0120-215/2019/4) and performed in accordance with the ethical standards laid down in the 1964 Declaration of Helsinki and its later amendments. All participants sign an informed consent form prior to their inclusion.

### 2.2 Laboratory methods

Peripheral blood samples were withdrawn at 3 time points: prior to TAVI, up to 24 h after TAVI and 3–6 months after TAVI on an ambulatory check-up. Blood was collected from the antecubital vein according to the standard procedure and collected into two 4 mL vacuum tubes that contained a coagulation activator and separating gel, as well as one vacuum tube containing K_3_-EDTA. Complete blood count with differential was measured in fresh EDTA blood. Serum was prepared by centrifugation at 2.000× g for 15 min. In fresh serum routine biochemistry parameters were determined (creatinine, high-sensitivity troponin I, NT-proB-type natriuretic peptide, total-, HDL- and LDL-cholesterol, creatin kinase and uric acid) by standard procedures. The remaining serum was aliquoted into plastic vials and stored at ≤−70°C until analysis. Concentrations of inflammatory biomarkers was assessed in thawed serum with Luminex's xMAP technology on a MagPix analyzer (R&D Systems, UK) according to the manufacturer's instructions.

### 2.3 Statistical analysis

Baseline characteristics are described as mean values and standard deviations for normally distributed or median and interquartile ranges for asymmetrically distributed data. Continuous variables were tested for normal distribution using the Shapiro–Wilk test. Categorical variables are expressed as frequencies and percentage. Comparisons between two groups were performed with t-test in case of normally distributed, with Mann–Whitney test for non-normally distributed data, and with chi-square test for proportions. Comparisons between more than two groups were performed using ANOVA.

For repeated measurements, data were analyzed using a linear mixed-effects model with a compound symmetry covariance structure, fitted via Restricted Maximum Likelihood (REML). This approach accounts for the correlation between repeated measurements and can handle missing values under the assumption that they are missing at random (MAR). In our study, a small number of biomarker measurements were missing (5% of total samples), distributed across baseline, 24 h, and 3–6 month time points. In the absence of missing data, results are equivalent to those obtained by repeated-measures ANOVA. Predictors were assessed using logistic regression to estimate odds ratios (ORs) with 95% confidence intervals (CIs), or linear regression to estimate regression coefficients (β) with standard errors (SEs). Survival analyses were performed using Kaplan–Meier curves and compared with the log-rank test. The impact of covariates on outcomes was assessed using Cox proportional hazards regression, reporting hazard ratios (HRs) with 95% CIs. No formal sample size calculation was performed, as this was an exploratory, hypothesis-generating study. To reduce the risk of overfitting and improve model interpretability given the small sample size and limited number of events, model simplification was applied. A two-sided *p*-value of <0.05 was considered statistically significant. Internal validation was performed using bootstrapping with 1,000 resamples to assesss model stability and potential overfitting. Regression coeficient and 95% confidence intervals were bias-corrected using the bootstrap procedure. Analyses were performed using GraphPad Prism (v10.2.3, GraphPad Software, LLC) and SPSS (v28.0.1.1, IBM Corp.).

## 3 Results

### 3.1 Baseline patients’ population

**Figure 1:**
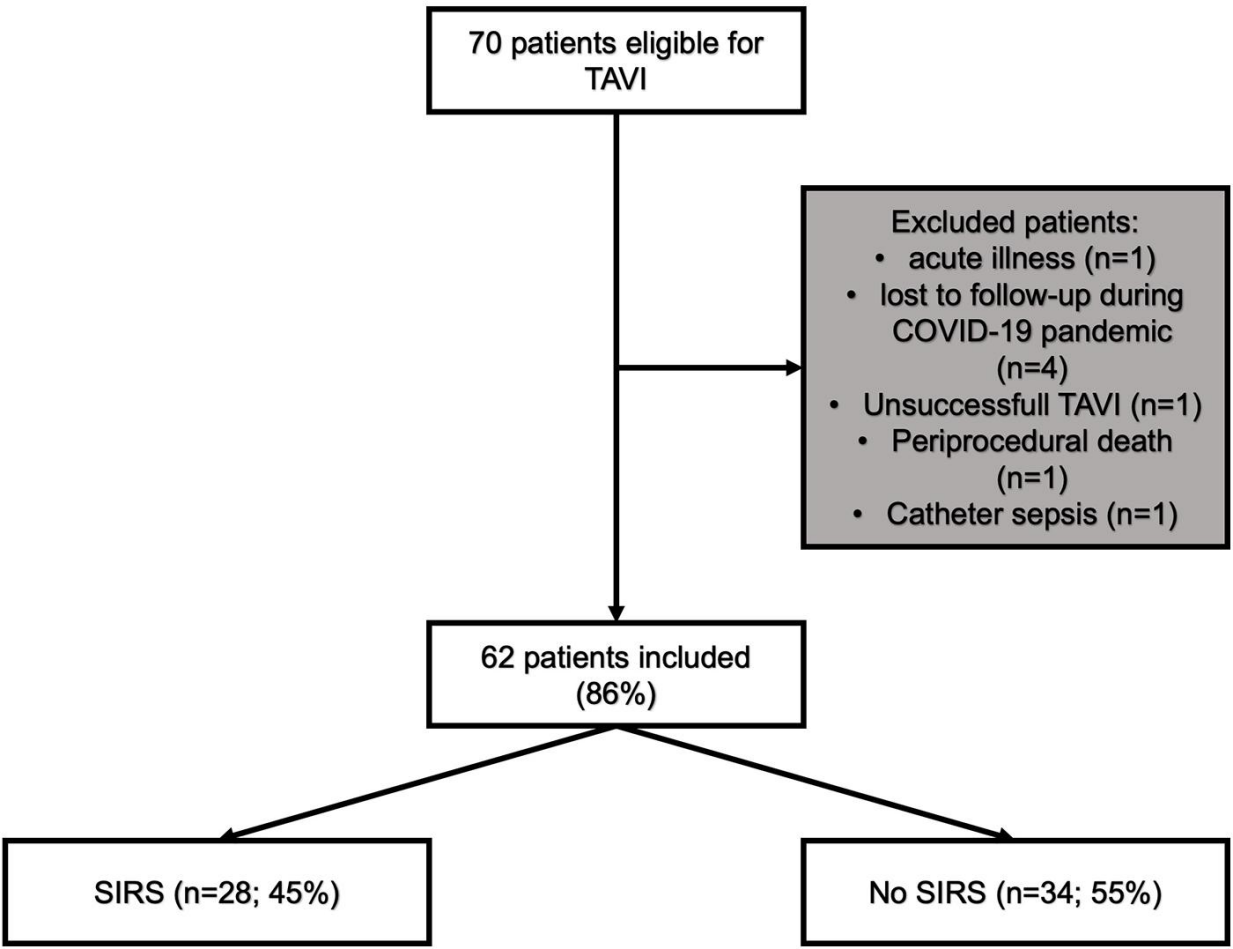
Flowchart of patients included in the study. COVID-19, coronavirus disease; SIRS, systemic inflammatory response syndrome; TAVI, transcatheter aortic valve implantation.

After excluding 8 patients, 62 were included into further analysis (Figure 1). Median group age was 80 (IQR: 76–84) years with the majority being female (56.5%). Median EuroSCORE II and STS score were 3.1 (IQR: 2.0–6.1) and 3.4 (IQR: 2.4–6.1) respectively. Diabetes mellitus was present in 15 (24%) and coronary artery disease in 32 (52%) patients. On admission, median leukocytes count was 7.0 × 10^9^ (IQR: 5.5–8.1 × 10^9^) /L and hs-CRP was 3 (IQR: 1.2–6.8) mg/L. Other baseline data is depicted in Table 1.

**Table 1:** Baseline clinical and imaging characteristics.

	SIRS patients (*n* = 28)	No SIRS patients (*n* = 34)	*p* value
Age, years	81 (75–84)	80 (76–83)	0.65
Gender, female	18 (64.3)	17 (50.0)	0.26
BMI, kg/m^2^	26.1 (23.0–30.4)	26.2 (23.7–31.4)	0.64
EuroSCORE II, %	2.6 (2.0–8.5)	3.4 (2.0–6.0)	0.82
STS score, %	3.2 (2.3–5.7)	4 (2.5–7.0)	0.42
Diabetes mellitus	7 (25.0)	8 (23.5)	0.89
Hypertension	26 (92.9)	31 (91.2)	0.81
Hyperlipidemia	20 (71.4)	31 (91.2)	**0**.**043**
Coronary artery disease	15 (53.6)	17 (50.0)	0.78
Peripheral artery disease	15 (53.6)	18 (52.9)	0.96
COPD	4 (14.3)	6 (17.7)	0.72
Previous PCI	6 (21.4)	4 (11.8)	0.30
Previous AMI	5 (17.9)	5 (14.7)	0.74
Previous stroke	2 (7.1)	0	0.11
Previous PM implantation	4 (14.3)	7 (20.6)	0.52
History of atrial fibrillation	10 (35.7)	11 (32.4)	0.78
RAAS inhibitors	23 (82.1)	24 (70.6)	0.29
Statins	16 (57.1)	29 (85.3)	**0**.**013**
Echocardiography:			
Left ventricular EF, %	52 ± 11	57 ± 15	0.23
Left ventricular EF <35%	1 (3.6)	3 (8.8)	0.40
Aortic valve maximal velocity, m/s	4.1 (3.7–4.5)	4.1 (4.0–4.5)	0.55
Aortic valve area, cm^2^	0.6 (0.5–0.8)	0.7 (0.6–0.8)	0.17
Aortic valve mean gradient, mmHg	44 ± 13	45 ± 13	0.80
Pulmonary hypertension	19 (67.9)	23 (67.7)	0.99

Data are presented as number (%), mean ± SD or median (IQR).

AMI, acute myocardial infarction; BMI, body mass index; COPD, chronic obstructive pulmonary disease; EF, ejection fraction; EuroSCORE, European System for Cardiac Operative Risk Evaluation score; PM, pacemaker; PCI, percutaneous coronary intervention; RAAS, Renin-angiotensin-aldosterone system; STS, Society of Thoracic Surgeons.

Statistically significant *p* values are presented in bold.

### 3.2 SIRS patients after TAVI

In the first 48 h after TAVI, SIRS developed in 28 (45%) patients. These patients were more likely to have elevated leukocyte count (35.7% vs. 2.9%; *p* < 0.001), tachycardia with a heart rate above 90 beats per minute (75.0% vs. 5.9%; *p* < 0.001) and hyperventilation with a respiratory rate of more than 20 per minute (96.4% vs. 52.9%; *p* < 0.001). When comparing baseline characteristics, patients with SIRS had less hyperlipidemia and statin prescriptions, higher levels of total cholesterol, LDL-cholesterol, and leukocytes. Other baseline characteristics where not statistically significant between the two observed groups (Tables 1, 2).

**Table 2:** Baseline laboratory findings.

	SIRS patients (*n* = 28)	No SIRS patients (*n* = 34)	*p* value
Creatinine, mmol/L	88 (65–113)	89 (76–117)	0.66
eGFR, mL/min/1.73 m^2^	60 ± 19	59 ± 20	0.71
Haemoglobin level, g/L	127 ± 20	127 ± 16	0.99
NT-proBNP, ng/L	3,147 (1,876–6,047)	2,730 (1,292–4,906)	0.33
Uric acid, mmol/L	405 ± 113	381 ± 117	0.43
Total cholesterol, mmol/L	4.1 ± 0.8	3.6 ± 0.8	**0**.**023**
HDL-cholesterol, mmol/L	1.2 ± 0.3	1.1 ± 0.3	0.51
LDL-cholesterol, mmol/L	2.4 ± 0.7	2.0 ± 0.7	**0**.**017**
Triglycerides, mmol/L	1.3 (0.9–1.7)	1.2 (0.9–1.8)	0.97
CK, μkat/L	1.3 (0.63–1.84)	1.61 (1.1–2.46)	0.08
hs-CRP, mg/L	3.4 (2.2–6.9)	1.9 (0.6–5.1)	0.078
Leukocyte count, ×10^9^ /L	7.5 (6.2–9.1)	6.5 (5.1–7.6)	**0**.**01**

Data are presented as number (%), mean ± SD or median (IQR).

CK, creatin kinase; eGFR, estimated glomerular filtration rate; HDL, high density lipoprotein; hs-CRP, high sensitivity C-reactive protein; LDL, low density lipoprotein; NT-proBNP, N-terminal B-type natriuretic peptide.

Statistically significant *p* values are presented in bold.

### 3.3 Predictors for SIRS after TAVI

When analyzing procedural characteristics, SIRS most frequently occurred in patients who experienced peri-procedural stroke, major vascular complication, and a greater drop in hemoglobin levels. Additionally, patients with SIRS more often required post-dilatation of the bioprosthetic aortic valve and had higher peri-procedural troponin levels. Their hospital stay was, on average, 2 days longer compared with patients without SIRS (Table 3).

**Table 3:** Procedural characteristics.

	SIRS patients (*n* = 28)	No SIRS patients (*n* = 34)	*p* value
Procedural access site: Transfemoral Transapical Transaortic	27 (96.4) 0 1 (3.5)	33 (97.1) 1 (3.9) 0	0.70
BEV	10 (35.7)	14 (41.2)	0.66
Valve-in-valve	2 (7.1)	3 (8.8)	0.81
Concomitant PCI	1 (3.6)	3 (8.8)	0.40
Pre-dilatation	16 (57.1)	19 (55.9)	0.92
Post-dilatation	12 (42.9)	6 (17.7)	**0**.**03**
Peri-procedural stroke	5 (17.9)	1 (2.9)	**0**.**048**
New pacemaker implantation	6 (21.4)	3 (8.8)	0.16
Major vascular complication	5 (17.9)	1 (2.9)	**0**.**048**
Acute kidney injury	6 (21.4)	4 (11.8)	0.30
At least moderate PVL	2 (7.1)	1 (2.9)	0.44
Blood transfusion	5 (17.9)	6 (17.7)	0.98
Drop in Hb levels, g/L	15.4 ± 6.9	10.3 ± 6.1	**0**.**006**
Hospitalization length, days	12 (7–20)	8 (5–19)	0.22
Length of stay after TAVI	7 (5–12)	5 (4–6)	**0**.**024**
Post-procedural aortic valve mean gradient, mmHg	8 (5–11)	9 (6–13)	0.33
Peri-procedural troponin levels	908 (323–2,415)	334 (157–890)	**0**.**039**

Data are presented as number (%), mean ± SD or median (IQR).

BEV, balloon expandable valve; Hb, hemoglobin; PCI, percutaneous coronary intervention; PVL, paravalvular regurgitation; TAVI, transcatheter aortic valve implantation.

Statistically significant *p* values are presented in bold.

All variables associated with the development of SIRS in the univariate regression analysis were considered for inclusion in the multivariable model. To minimize overfitting and maintain model stability [events-per-variable ratio (EPV) = 5.6], only baseline laboratory parameters—leukocyte count, LDL-cholesterol, and hs-CRP ≥ 2 mg/L—were included in the multivariable analysis. After adjustment, all variables showed a trend towards association with the development of SIRS but did not reach statistical significance (See Supplementary Table S1).

### 3.4 Novel biomarkers

Serum levels of hs-CRP, sST2/IL33, IL-10 and IL-2 showed significant changes after TAVI, with initial increases followed by subsequent decreases during follow-up. Overall, hs-CRP levels at follow-up were significantly lower compared to baseline. There was no significant rise in TNF-α after the procedure, however, values at follow-up were lower than pre-procedure or immediately after. Detailed statistical results are presented in Supplementary Table S2.

**Figure 2:**
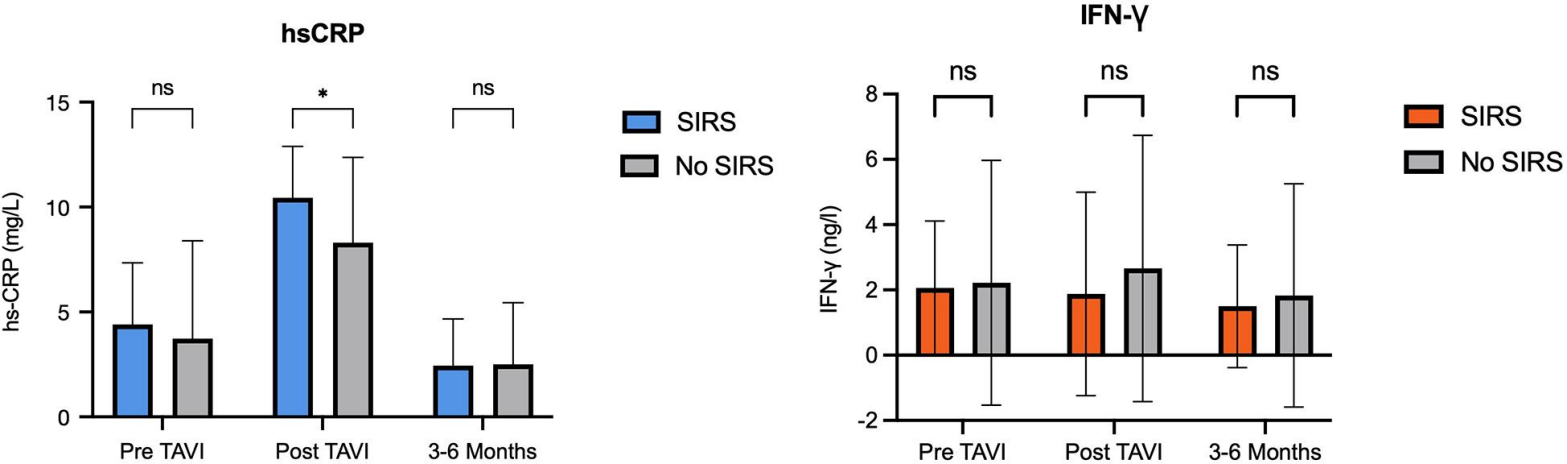
Levels of hs-CRP and IFN-γ in patients with and without SIRS. hs-CRP, high sensitivity C-reactive protein; IFN, interferon; SIRS, systemic inflammatory response syndrome.

When comparing biomarker dynamics between groups, only post-TAVI hs-CRP levels were significantly higher in patients who developed SIRS [mean difference 2.1 (95% CI: 0.4; 3.9); *p* = 0.02] (Figure 2). After adjustment, age (−0.2, SE 0.067; *p* = 0.005) and periprocedural stroke (3.69, SE 1.53; *p* = 0.019) were independently associated with greater increase in hs-CRP levels after TAVI. No other independent predictors were identified for the remaining biomarkers.

### 3.5 Predictors for outcome in patients with SIRS after TAVI

Median follow up time of patients in our study was 2.9 (IQR: 1.1–5.0) years. No patients included in the study died at 30 days or 6 months. Three patients out of 62 (4.8%) died during the first year, five out of 40 (12.5%) at 2 years, five out of 35 (17.5%) at 3 years, three out of 30 (10%) at 4 years and two out of 43 (4.7%) at 5 years. Two out of 62 (3.2%) patients where re-hospitalized for any unplanned cause at 30 days, seven out of 60 (11.7%) at 6 months, three out of 53 (5.7%) at 1 year, seven out of 30 (23.3%) at 2 years, five out of 23 (21.7%) at 3 years, one out of 16 (6.3%) at 4 year and four out of 13 (30.8%) at 5 years. Causes for unplanned rehospitalization were heart failure (*n* = 9), pacemaker implantation (*n* = 4) and sepsis (*n* = 5), gastrointestinal bleeding (*n* = 4), acute coronary syndrome (*n* = 3), COVID-19 infection (*n* = 3), intracranial bleeding (*n* = 1), and malignancy (*n* = 1).

**Figure 3:**
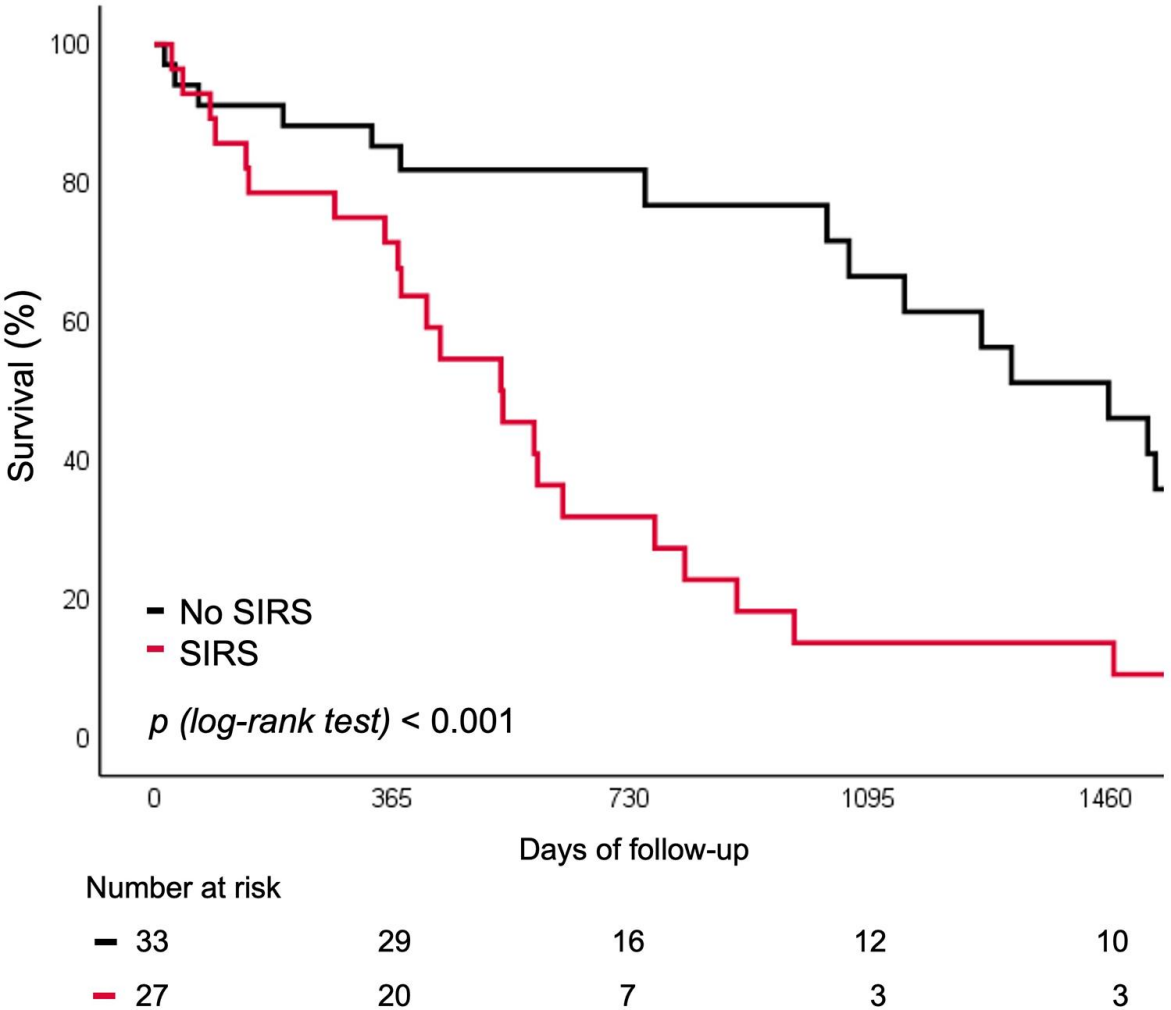
Survival curve for rehospitalization or death for any cause according to SIRS. SIRS, systemic inflammatory response syndrome.

Patients who developed SIRS following TAVI exhibited a markedly higher incidence of all-cause death or unplanned rehospitalization at 5 years compared with those without SIRS (35.8% vs. 4.5%; *p* < 0.001) (Figure 3). This association persisted after exclusion of patients with peri-procedural stroke (*n* = 6), major vascular complications (*n* = 6), or surgical access (*n* = 2) that could have confounded long-term outcomes (41.7% vs. 0%; *p* = 0.001).

In univariate Cox regression analysis, patients with a major vascular complication had an almost six-fold increased risk of unplanned rehospitalization or death from any cause over the 5-year follow-up (HR 5.95, 95% CI 2.23–25.58; *p* < 0.001). A three-fold increased risk was observed in patients with SIRS (HR 3.07, 95% CI 1.57–6.00; *p* = 0.001). In the multivariable model, only the two biomarkers and the STS score were included based on clinical relevance and to maintain model stability (EPV = 7.6). SIRS and major vascular complications were reported in univariable analyses but were excluded from the multivariable model to avoid overfitting. After adjustment, baseline hs-CRP (HR 1.17, 95% CI 1.04–1.31; *p* = 0.008) and IFN-γ (HR 1.18, 95% CI 1.03–1.34; *p* = 0.015) were independently associated with freedom from any rehospitalization or death after TAVI (Table 4). Internal validation yielded a bias-corrected 95% confidence intervals crossing 1, reflecting uncertainty due to the small sample size; however, bootstrap-corrected *p*-values remained significant, supporting stability of this associations (Supplementary Table S3).

**Table 4:** Cox uni- and multivariable regression analysis for unplanned hospitalization and death for any cause.

	Univariate analysis			Multivariable analysis	
	HR [95% CI]	*p* value		HR [95% CI]	*p* value
Age	0.99 [0.95; 1.04]	0.846			
Sex (female)	0.63 [0.33; 1.22]	0.170			
BMI	0.98 [0.91;1.06]	0.574			
SIRS	3.07 [1.57; 6.0]	**0**.**001**			
STS score	1.07 [1.0; 1.15]	**0**.**049**	STS score	0.82 [0.91; 1.08]	0.823
Valve type	0.53 [0.27; 1.05]	0.07			
Coronary artery disease	1.5 [0.80; 2.93]	0.20			
Major vascular complications	5.95 [2.23; 15.58]	**<0**.**001**			
Baseline hs-CRP	1.21 [1.09; 1.35]	**<0**.**001**	Baseline hs-CRP	1.17 [1.04; 1.31]	**0**.**008**
Baseline IFN-γ	1.22 [1.09; 1.36]	**<0**.**001**	Baseline IFN-γ	1.18 [1.03; 1.34]	**0**.**015**

BMI, body mass index; hs-CRP, high sensitivity C-reactive protein; IFN, interferon; SIRS, systemic inflammatory response syndrome; STS, society of thoracic surgeons; TAVI, transcatheter aortic valve implantation.

Statistically significant *p* values are presented in bold.

**Figure 4:**
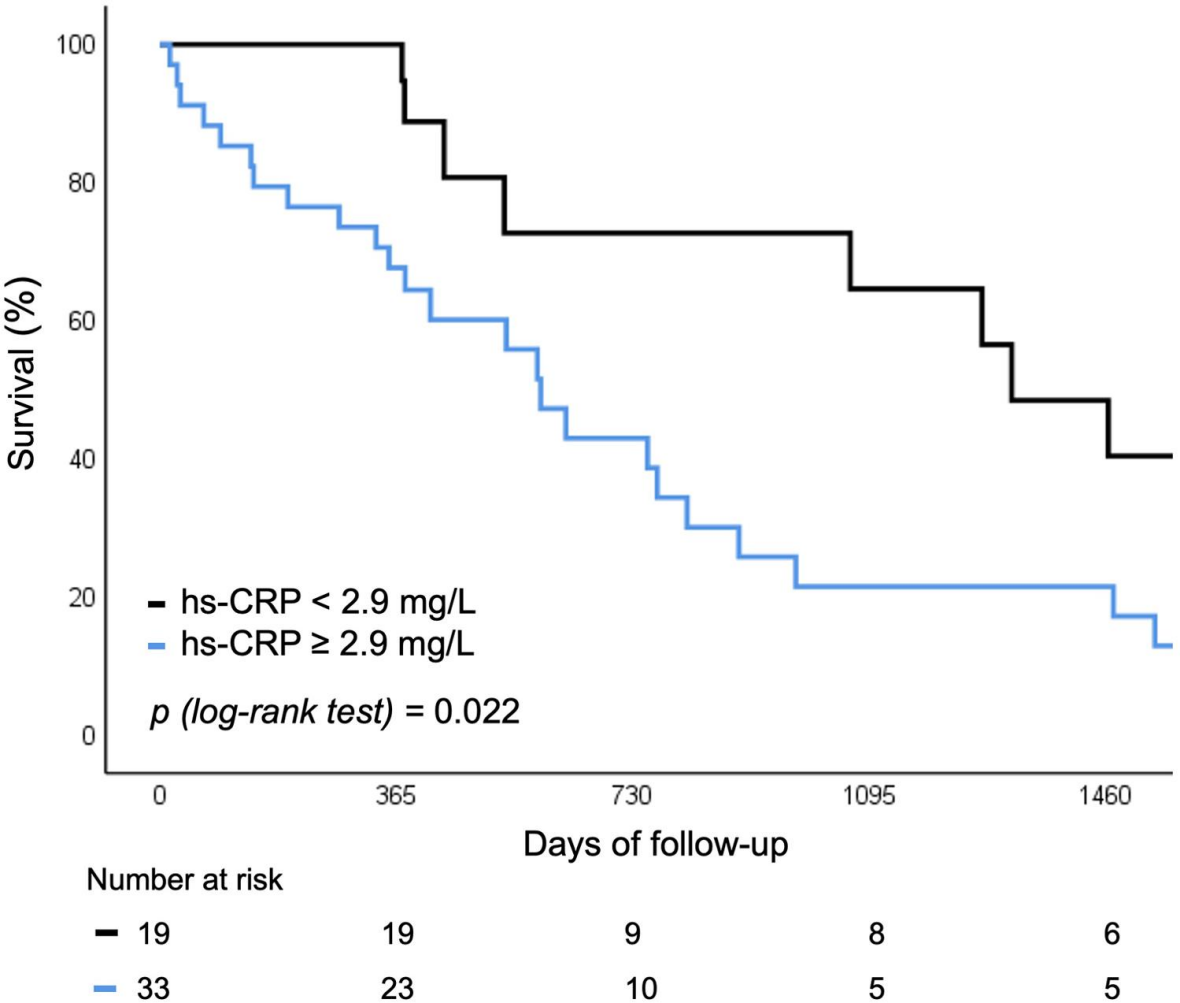
Survival curve for rehospitalization or death for any cause according to baseline hs-CRP levels. hs-CRP, high sensitivity C-reactive protein.

**Figure 5:**
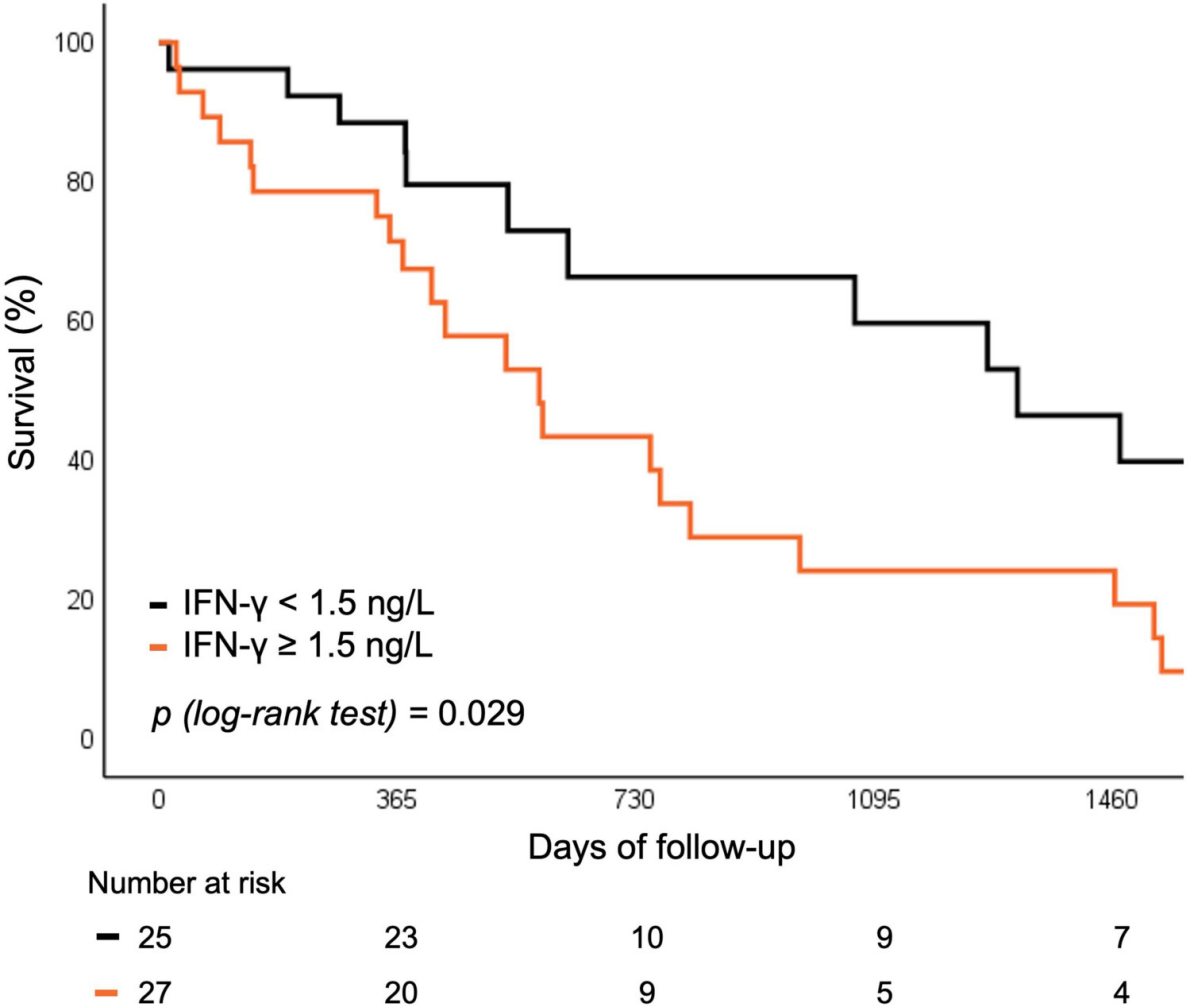
Survival curve for rehospitalization or death for any cause according to baseline IFN-*γ* levels. IFN, interferon.

Baseline levels of hs-CRP and IFN-γ above vs. below the median were predictive of the risk of rehospitalization or all-cause mortality. Both biomarkers were associated with worse 5 year outcomes, independent of SIRS development (12.9% vs. 32.3%; *p* = 0.022 for hs-CRP and 9.6% vs. 33.2%; *p* = 0.029 for IFN-γ respectively) (Figures 4, 5).

## 4 Discussion

Our study has shown that TAVI is associated with an unfavorable inflammatory response—both in terms of a relatively high prevalence of SIRS and in terms of a transient increase in inflammatory biomarkers, irrespective of patient characteristics or procedure-related factors. Importantly, elevated baseline levels of hs-CRP and IFN-γ were independently associated with increased all-cause mortality and re-hospitalizations during long-term follow up, suggesting that these biomarkers may have a potential value for pre-procedural risk stratification in patients undergoing TAVI.

A systemic inflammatory response to TAVI was reflected by a substantial—i.e., 45% –prevalence of SIRS in our study. This is in line with previous studies reporting peri-procedural SIRS in 6%–56% patients undergoing TAVI (6–8, 19–21), and reflects pathophysiologic responses to the procedure, such as suboptimal organ perfusion and related tissue damage with cytokine release (22–24). Patients with SIRS in our study exhibited elevated leukocyte count, tachycardia and hyperventilation (as per SIRS definition). Interestingly, patients on baseline statin therapy were less likely to develop SIRS. While the causative mechanism of this association cannot be explained by our observational study design, statin therapy exerts anti-inflammatory pleiotropic effects (25), which may explain the reduced risk of SIRS in patients taking statins peri-procedurally.

Procedural steps—such as type of aortic valve, rapid pacing, post-dilatation and procedural complications—can all influence the development of SIRS (7, 8, 26). The onset of SIRS can be explained by hemodynamic and traumatic derangements. While the former may result from transient hypotension and hypoperfusion during procedural maneuvers, the latter may result from blunt forces directed to the aortic annulus culminating in release of cytokines during balloon valvuloplasty or valve implantation. Our study supports these mechanisms by identifying a relation between certain procedural characteristics (i.e., peri-procedural stroke, major vascular complications, drop in hemoglobin levels, post-dilatation) and the development of SIRS. This was further supported by significantly higher periprocedural troponin levels that are known to have a detrimental impact on short- and long-term prognosis in patients after TAVI (27, 28). As some of these complications can be reduced, efforts should be made to better utilize procedures with proved efficacy (such as ultrasound-guided vascular access) (29, 30), and reduce unnecessary steps causing transient hypotension or stroke (such as post-dilatation) (31). Moreover, our study showed SIRS patients experience longer post-procedural hospitalization periods. Better knowledge and detection could therefore additionally impact procedural costs and reduce unnecessary hospital infections (32).

In addition to SIRS, TAVI is associated with a subclinical inflammatory response, as reflected by dynamic changes in serum biomarkers. Levels of hs-CRP, sST2/IL-33, IL-10 and IL-2 changed significantly in all patients undergoing TAVI, irrespective of clinically detectable SIRS. Significant rise in leukocyte count, IL-6, IL-8 and hs-CRP levels have already been described in the literature (7, 9, 19). Our study adds to this knowledge by demonstrating important dynamics of TNF-α, sST2/IL-33, IL-10 and IL-2.

The gradual decrease of TNF-α after TAVI supports the hypothesis of an underlying subclinical inflammation in patients with severe aortic stenosis. TNF-α is a precursor of IL-6, a pro-inflammatory cytokine involved in tissue inflammation and valve calcification (33). Thus, TAVI not only improves central cardiac hemodynamics, as reflected by an improvement in systemic endothelial function (34), but may also mitigate underlying inflammation. While the rise of both anti-inflammatory IL-10 and pro-inflammatory IL-2 likely reflects a balanced inflammation process during implantation, the dynamic of sST2/IL-33 levels add knowledge to the tissue damage hypothesis by promoting tissue fibrosis. Among all biomarkers, post-procedural hs-CRP was the only marker distinguishing patients with and without SIRS, likely reflecting procedural characteristics such as complications.

Importantly, the occurrence of SIRS and baseline levels of hs-CRP and IFN-γ were associated with long-term outcomes after TAVI. Previous studies have reported inconsistent findings—showing either worse (7, 19) or similar (8) event rates in relation to SIRS. In our study, these association persisted after adjustment for procedural complications and access site, suggesting that novel blood biomarkers may aid in pre-procedural risk stratification. While baseline hs-CRP and Th2 cell levels have previously been linked to 1-year mortality (9), this is, to our knowledge, the first study identifying baseline hs-CRP and IFN-γ as independent predictors in TAVI patients.

The prognostic relevance of CRP is not unique to TAVI and has also been demonstrated in other cardiovascular conditions. In patients with acute myocardial infarction complicated by cardiogenic shock, higher baseline CRP levels were independently associated with increased 30-day all-cause mortality (35). Similarly, in a larger cohort of patients with cardiogenic shock, admission CRP levels strongly predicted both 30-day and 1-year mortality, with patients in the highest quartile demonstrating more than a two-fold higher risk compared to those in the lowest quartile (36). Moreover, incorporating CRP levels into established cardiogenic shock risk scores improved their predictive accuracy (37), underscoring the central role of systemic inflammation across different cardiac pathologies.

Circulating hs-CRP has also been associated with increased risk of coronary artery disease, ischemic stroke, and heart failure (38, 39). Measurement of baseline hs-CRP may therefore serve as a risk-stratification marker, and could guide future research into potential interventions. While anti-inflammatory therapies such as statin therapy (25), colchicine (40, 41), or canakinumab (42) have shown effects on IL-6 and CRP levels in other populations, their benefit in TAVI setting remains speculative. The overlap in inflammatory profiles between patients with atherosclerosis and those undergoing TAVI suggests an imbricated pathophysiologic mechanism, but further studies are needed before any conclusions about therapeutic strategies can be drawn.

Levels of IFN-γ have already been attributed an important role in connection with all stages of atherosclerosis and heart failure (14, 43, 44). While some studies report increased levels to be associated with atherogenesis, myocardial inflammation, hypertrophy and fibrosis, others report an opposite protective effect against hypertrophy and diastolic dysfunction (15, 43). So far, IFN-γ pathways blockage has not shown beneficial effects on the cardiovascular system. Its potential role as target for future treatment is therefore yet to be fully elucidated.

While TAVI implantations are expanding in the younger population, structural valve deterioration remains of great concern due to limited bioprosthetic valve durability and potential need for further invasive procedures (45). Knowing inflammation plays a crucial role in this processes (46), future detection of specific inflammatory biomarkers could represent an important tool for patient monitoring.

In summary, patients with severe aortic stenosis appear to have a pre-existing subclinical inflammatory state that may adversely affect survival after TAVI. The clinical relevance of this finding and its potential implications for targeted therapy should be confirmed in larger studies with greater statistical power.

### 4.1 Study limitations

Our study has been conducted on a relatively small, single center cohort and should therefore be regarded as hypothesis-generating. Although we prospectively enrolled consecutive patients and used standardized procedures, selection bias and residual confounding cannot be fully excluded. Serial biomarker measurements in larger, multicenter TAVI populations are needed to validate our findings. Nonetheless, the results of this prospective study align with existing literature and may serve as a foundation for developing improved criteria for defining SIRS after TAVI, potentially incorporating novel biomarkers to better predict patient outcomes.

## 5 Conclusion

Systemic inflammatory response syndrome is a frequent finding after TAVI, likely reflecting procedural stress, tissue injury, and associated inflammatory activation. Elevated levels of hs-CRP and IFN-γ before TAVI were associated with a higher risk of adverse long-term outcomes, suggesting their potential exploratory role in identifying patients who may benefit from closer follow-up.

## Data Availability

The raw data supporting the conclusions of this article will be made available by the authors, without undue reservation.
