# Supplementary material for: Inflammatory biomarkers as predictors of prognosis in patients after transcatheter aortic valve implantation
Source: Front Cardiovasc Med. 2026 Jan 2;12:1722293. doi: 10.3389/fcvm.2025.1722293 (PMC12808394; doi:10.3389/fcvm.2025.1722293)
Supplement: Supplementary file 1 [file Datasheet1.docx]

Supplementary Material

**Supplementary Table 1** Uni and multivariable regression analysis for development of SIRS after TAVI

|  | **Univariate analysis** | |  | **Multivariable analysis** | |
| --- | --- | --- | --- | --- | --- |
|  | **OR [95% CI]** | ***p* value** |  | **OR [95% CI]** | ***p* value** |
| Age | 1.01 [0.94; 1.1] | 0.80 |  |  |  |
| Sex (female) | 1.8 [0.65; 5.13] | 0.26 |  |  |  |
| BMI | 0.97 [0.88; 1.08] | 0.57 |  |  |  |
| Post-dilatation | 2.89 [0.945; 8.85] | 0.06 |  |  |  |
| Major vascular complications | 7.17 [0.79; 65.53] | 0.08 |  |  |  |
| Drop in Hb levels | 1.10 [1.03; 1.18] | **0.008** |  |  |  |
| Baseline leukocytes count | 1.44 [1.08; 1.91] | **0.012** | Baseline leukocytes count | 1.37 [0.98; 1.93] | 0.07 |
| LDL-cholesterol | 2.53 [1.13; 5.63] | **0.023** | LDL-cholesterol | 2.55 [0.88; 7.37] | 0.08 |
| Statins | 0.23 [0.07; 0.77] | **0.017** |  |  |  |
| Baseline hs-CRP ≥2 mg/L | 4.29 [1.26; 14.53] | **0.019** | Baseline hs-CRP ≥2 mg/L | 3.15 [0.82; 12.08] | 0.10 |

^BMI = body mass index; hs-CRP = high sensitivity C-reactive protein; LDL = low density lipoprotein; TAVI = transcatheter aortic valve implantation.^

^Statistically significant^ *^p^* ^values are presented in bold.^

**Supplementary Table 2** Novel biomarkers dynamics in all patients undergoing TAVI

|  | **Mean diff**  **(pre vs. post)** | ***p* value** | **Mean diff**  **(pre vs. FU)** | ***p* value** | **Mean diff**  **(post vs. FU)** | ***p* value** |
| --- | --- | --- | --- | --- | --- | --- |
| hs-CRP, mg/L | 5.2 [3.8; 6.6] | **<0.001** | -1.6 [-2.8; -0.33] | **0.01** | - 6.8 [-8.0; -5.6] | **<0.001** |
| TNF-α, ng/L | -3.5 [-16.7; 9.7] | 0.80 | -15.4 [-25.7; -5.0] | **0.002** | -11.9 [-19.5; -4.3] | **0.001** |
| sST2/IL-33, μg/L | 17129 [5958: 28300] | **0.002** | -2136 [-5695; 1424] | 0.33 | -19265 [-31526; -7003] | **0.001** |
| IL-10, ng/L | 1.9 [0.5; 3.3] | **0.005** | -0.4 [-1.1; 0.4] | 0.47 | -2.3 [-3.5; -1.0] | **<0.001** |
| IL-1β, ng/L | 0.4 [-0.2; 1.0] | 0.18 | 0.002 [-0.006; 0.003] | 0.57 | -0.4 [-1.0; 0.1] | 0.14 |
| IFN-γ, ng/L | 0.2 [-0.5; 0.8] | 0.80 | -0.5 [-1.6; 0.7] | 0.61 | -0.6 [-1.8; 0.6] | 0.43 |
| IL-2, ng/L | 0.7 [0.3; 1.1] | **<0.001** | -0.3 [-0.6; 0.004] | 0.05 | -1.0 [-1.5; -0.5] | **<0.001** |

^hs-CRP = high sensitivity C-reactive protein; IFN = interferon; IL = interleukin; sST = soluble suppression of tumorogenesis; TNF = tumor necrosis factor.^

^Statistically significant^ *^p^* ^values are presented in bold.^

**Supplementary Table 3** Cox multivariable regression model validated by bootstrapping

|  | **Multivariable analysis** | | **Bootstrapping** | |
| --- | --- | --- | --- | --- |
|  | **HR** | ***p* value** | **Bootstrap 95% CI** | **Boostrap *p* value** |
| STS score | 0.99 | 0.82 | -0.17; 0.15 | 0.83 |
| Baseline hs-CRP | 1.17 | **0.008** | -0.03; 0.33 | **0.009** |
| Baseline IFN-γ | 1.18 | **0.015** | -0.17; 0.34 | **0.014** |

^hs-CRP = high sensitivity C-reactive protein; IFN = interferon; STS = Society of Thoracic Surgeons.^

^Statistically significant^ *^p^* ^values are presented in bold.^
